# Maternal Protein Restriction Induces Alterations in Hepatic Unfolded Protein Response-Related Molecules in Adult Rat Offspring

**DOI:** 10.3389/fendo.2018.00676

**Published:** 2018-11-20

**Authors:** Xiaomei Liu, Jun Wang, Linlin Gao, Yisheng Jiao, Caixia Liu

**Affiliations:** ^1^Key Laboratory of Maternal-fetal Medicine of Liaoning Province, Shengjing Hospital, China Medical University, Shenyang, China; ^2^Department of Obstetrics and Gynecology, Benxi Central Hospital of China Medical University, Benxi, China; ^3^Medical Research Center, Shengjing Hospital, China Medical University, Shenyang, China; ^4^Department of Obstetrics and Gynaecology, Shengjing Hospital of China Medical University, Shenyang, China

**Keywords:** intrauterine growth restriction, unfolded protein response, hepatic, glucogenesis, rat models

## Abstract

Intrauterine growth restriction (IUGR) leads to the development of metabolic syndrome in adulthood. To explore the potential mechanisms of metabolic imprinting, we investigated the effect of malnutrition *in utero* on hepatic unfolded protein response (UPR)-related genes in IUGR offspring. An IUGR rat model was developed by feeding a low-protein diet to pregnant rats. The expression levels and activity of hepatic UPR genes were analysed by quantitative PCR (qPCR) arrays and western blotting. The hepatic UPR molecules heat-shock 70-kDa protein 4l (*Hspa4l*), mitogen-activated protein kinase 10 (*Mapk10*), and endoplasmic reticulum to nucleus signalling 2 (*Ern2*) were markedly downregulated in IUGR foetuses, but the expression of *Mapk10* and *Ern2* returned to normal levels at 3 weeks postnatal. In contrast, cAMP responsive element binding protein 3-like 3 (*Creb3l3*) was upregulated in hepatic tissues at embryo 20(E20), then restored to normal in adulthood (12 weeks). The protein levels of activating transcription factor 2 (Atf2) and Atf6, two key factors of the UPR pathway, were upregulated in the livers of IUGR foetuses, and the latter remained upregulated until 12 weeks. Combined with our previous findings showing an increase in hepatic gluconeogenesis enzymes in IUGR offspring, we speculated that aberrant intrauterine milieu impaired UPR signalling in hepatic tissues; these alterations early in life might contribute to the predisposition of IUGR foetuses to adult metabolic disorders.

## Introduction

Intrauterine growth restriction (IUGR) occurs in about 3–10% of pregnancies, and is considered one of the biggest causes of prenatal morbidity and mortality (1, 2). Multiple epidemiological studies have shown that a decline in foetal growth rates reflects an adaptation to an adverse environment *in utero*, and this may lead to persistent changes in metabolism, growth, and organ development (3, 4). Concerns over the programming effects of IUGR in the liver have gained momentum in recent years. Among US adolescents, the prevalence of suspected non-alcoholic fatty liver disease(NAFLD) has increased dramatically over the last two decades, now affecting approximately 11% of adolescents (5). Studies in birth cohorts show that low birth weight followed by rapid postnatal weight gain is positively associated with subsequent obesity and NAFLD, the latter is independent of insulin resistance (6–8). Using a well-defined IUGR model in rats by maternal protein restriction, our group previously demonstrated that IUGR animals developed glucose intolerance and hyperglycaemia in adulthood (9); this was closely related to disorders in cholesterol metabolism and glucogenesis (10), similar with other experimental model report (11). Despite the clearly demonstrated hepatic dysfunction in IUGR offspring, the underlying mechanisms of the programming effects on foetal livers remain unknown.

The endoplasmic reticulum (ER) is an important site of biosynthesis metabolism in eukaryotic cells, and also regulates post-translational protein processing and transport. Under some cellular stress conditions, such as malnutrition and lipid overload, protein folding disorders can occur. Unfolded/misfolded proteins accumulate in the ER lumen and trigger a cytoprotective response known as the ER stress response (ERSR) or unfolded protein response (UPR) (12, 13). The UPR is a complicated process, consisting of three signalling pathways and many factors that function by triggering the PKR-like ER kinase (PERK), activating transcription factor 6 (ATF6) and inositol-requiring protein-1 (IRE1) signalling pathways (14–16). In addition to these classical ER stress transducers, the cAMP-responsive element binding protein 3 (CREB3) and CREB3-like (CREB3L) 1–4 transcription factors elicit UPR signalling in a cell type- and context-specific manner (17). Once activated, UPR initiates protective responses, triggers the production of ER molecular chaperones and stress response proteins to enhance protein degradation, and increases the protein folding capacity of the ER (12). However, sustained ER stress ultimately leads to apoptotic cell death (18). If the burden of protein load in the ER is beyond its processing capacity, UPR can activate pro-apoptotic or autophagic pathways, leading to cell death.

Studies in both humans and animal models have confirmed that ER stress is closely linked to a variety of metabolic syndromes, including diabetes and non-alcoholic fatty liver disease. It was reported that ER stress can cause pancreatic β-cell dysfunction and apoptosis, as well as peripheral tissue insulin resistance; the ER stress response can also activate stress signalling pathways, cause hepatocyte cell death, and eventually lead to liver dysfunction (19–21).

Recently, the UPR pathway was reported to be associated with maternal programming. Maternal obesity and obesogenic diets post-partum in offspring triggered altered UPR signalling pathway in hepatic and pancreas, subsequent development of NAFLD and non-alcoholic fatty pancreas (22, 23). Graham et al. found that in the placenta of mice with eukaryotic initiation factor 2α (eIF2α)-overexpression, UPR was enhanced, impairing placental function and leading to the development of IUGR (24); UPR factors were also enhanced in adipose tissues of foetuses with IUGR caused by a uterine artery clamp. Riddle et al. (25) reported that components of the UPR (heat shock protein a5 [Hspa5], ATF6, and p-eIF2) were increased in the retroperitoneal adipose tissues of 3-week-old (3 W) IUGR rats induced by ligation, which contributed to the development of glucose intolerance in male IUGR rats.

Thus, evidence exists to support the working hypothesis that long-term modifications in the expression of key factors of UPR signalling pathways involved in glucose metabolism are associated with glucose intolerance in aging IUGR offspring. The present study investigated the impact of IUGR, induced through maternal protein restriction, on the expression of components of the UPR signalling pathway. This work aimed to elucidate the development of hyperglycaemia and related underlying patterns of gene expression.

## Experimental methods

### Animals and tissue collection

**Figure 1:**
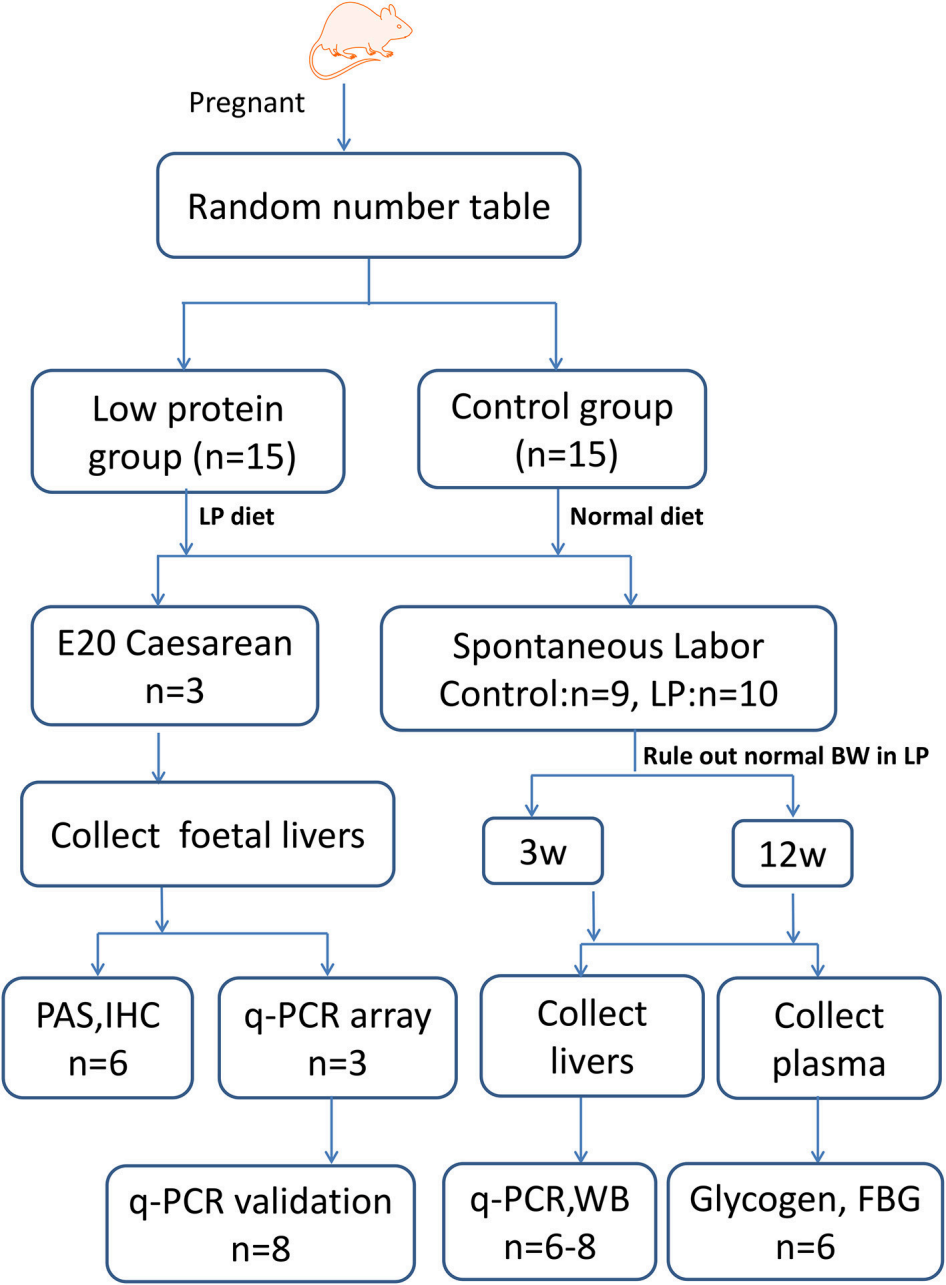
Experimental design. Timed-pregnant Wistar rats were fed a low-protein diet (undernourished group) or normal diet (control group) from day 0 of pregnancy until full term. A subset of rats was killed at E20 and foetal livers were collected for qPCR array, periodic acid-Schiff (PAS) and immunohistochemical (IHC) staining, and western blotting (WB) studies. Another subset of offspring was delivered spontaneously, male offspring were sacrificed at 3 and 12 W, and the median lobes of their livers were collected for WB and qPCR analysis. LP, low protein; BW, birth weight; IUGR, intrauterine growth restriction; FBG, fast blood glucose.

All animal study protocols were approved by the Animal Research Committee of China Medical University (ethics approval number: 2015PS41K). All the research below adhered to Laboratory Biosecurity Guidance of China Medical University. Adult Wistar rats were housed under specific pathogen-free conditions in an environmentally-controlled clean room at the Experimental Animal Centre (Shengjing Hospital, China Medical University). All animals were individually housed in polypropylene cages with a wire mesh top and a hygienic bed of husk under controlled conditions of a 12 h light/dark cycle, at a temperature of 22 ± 2°C and relative humidity of 40–60% throughout the experimental period. The experimental design is summarized in Figure 1.

Model rats establishment experiments were performed as described previously (10). Briefly, timed-pregnant Wistar rats (10–12 weeks) weighing 230–260 g were randomly divided into two groups according to the random number table method (*n* = 15 per group): animals in the undernourished group received an isocaloric low-protein diet (7% protein; Beijing HFK Bioscience, Beijing, China) from day 0 of pregnancy until full term; control animals were maintained with a standard diet (22% protein) during gestation. The composition and nutrition levels of the diets are shown in Table 1. A foetus with a body weight of two standard deviations less than the mean body weight of the control group was classified as IUGR, according to the definition of human IUGR (26). On day 20 of gestation (E20, near-term foetuses), a set of pups (three litters per group) was delivered by caesarean section. On the basis of body weight, foetal blood was pooled from control or IUGR foetuses (n = 3) within a litter to quantify glucose levels. Three IUGR foetuses (average body weight 3.11 ± 0.31 g, *n* = 22) and three control foetuses (average body weight 4.43 ± 0.30 g, *n* = 33) were obtained from each of the six litters, and the median lobes of foetal livers were collected. Another subset of rats was allowed to deliver spontaneously and litters of 8–12 pups were selected for subsequent experiments, to exclude the effects of unbalanced development caused by litter size. One litter of new-born rats from the control group were excluded because the litter size exceed twelve. The remaining pups were nourished by their mothers until weaning at 3 weeks postnatal (3 W). The offspring rats whose birth weight does not meet the IUGR criteria were sacrificed after anesthesia and only IUGR offsprings were fed under normal conditions until 24 weeks (24 W). The rat offspring were given access to water and feed *ad libitum*, and the remaining diet was collected and weighed each morning. Body weight was recorded weekly. Food intake was determined daily at 3 W, 8 weeks (8 W), and 12 weeks (12 W). Eight pups from each group were killed under ether anaesthesia at 3 and 12 W; the median lobes of their livers were excised, rinsed in saline, and frozen at −80°C until analysis. To avoid any interference due to sex and hormonal differences, the following analyses were performed on male pups only, except foetal analyses.

**Table 1:** Composition of rat diets.

	**Standard diet (%)**	**LP diet (%)**
Protein	22.2	7
Fat	4.8	6.9
Carbohydrates	61.2	74.7
Ca	1.62	0.53
P	0.92	0.23
Calories	399 kcal/100 g	395 kcal/100 g

*LP, low protein*.

### Biochemical analysis

Fasting blood glucose was measured at 3 and 12 W. Liver glycogen concentration was measured at the same timepoints by a colorimetry assay using a liver glycogen test kit (Jiancheng, Nanjing, China) according to the manufacturer's protocol, and the result was expressed as mg glycogen/g wet weight liver tissue.

### Liver histology staining

Fresh liver tissues were fixed in 4% paraformaldehyde for 48 h before processing using an automatic tissue processor for paraffin embedding. Embedded tissues were sliced into 3-μm sections, and the sections were deparaffinized using xylene, xylene/ethanol, and ethanol, and then hydrolysed with water. Periodic acid-Schiff (PAS) staining was performed using the AB-PAS staining kit (Solarbio, Beijing, China) according to the manufacturer's instructions. The sections were incubated with alcian blue solution for 10 min, washed with ddH_2_O, stained with Schiff reagent for 20 min, washed again with ddH_2_O, and then stained with haematoxylin for 1 min. The sections were washed with ddH_2_O, dehydrated, and mounted onto slides using a neutral resin. Additional sections were subjected to immunohistochemical (IHC) staining according to standard procedures with primary antibodies. The primary antibodies used for IHC staining included rabbit anti-heat shock protein A4L (HSPA4L) (1:100, orb184242, Biorbyt, Cambridge, United Kingdom), anti-CREB3L3 (1:300, YN1809, Immunoway, Plano, TX, United States), and anti-mitogen activated protein kinase 10 (MAPK10) (1:100, E1A5321, Enogene, Nanjing, China). A negative control without the primary antibody was also included and no unwanted background staining was observed. Imaging was performed under high magnification (400×) using a Nikon ECLIPSE Ti microscope. All images were analysed with NIS-Elements BR 2.10 image analysis system (Nikon, Tokyo, Japan). The integral optical density (IOD) under each examined field in each group was determined, and the average glycogen content was calculated and analysed.

### Quantitative PCR (qPCR) array

The mRNA levels of ninety-six genes (84 UPR-related genes, five housekeeping genes, and seven quality control genes; Supplementary Table 1; a complete list of genes is also available at http://www.sabiosciences.com/rt_pcr_product/HTML/PAHS-013A.html) were measured using qPCR arrays (cat #: PAHS-013A; SA Biosciences, Frederick, MD, United States). To reduce individual variation, nine controls from three litters were pooled into 3 samples as control1, control2, and control3, and nine IUGRs were pooled into 3 samples as IUGR1, IUGR2, and IUGR3. Total RNA was extracted using the RNeasy Plus Mini Kit (Qiagen, Hilden, Germany) according to the manufacturer's instructions, followed by DNase I digestion. The concentration and purity of RNA were checked in duplicate using a spectrophotometer (IMPLEN NP80, Munich, Germany); a ratio of A260/280 between 1.8 and 2.0 was obtained for all RNA samples. The integrity of ribosomal RNA was detected through capillary gel electrophoresis using the QIAxcel Advanced System. The cDNA was then synthesized using an RT^2^ First Strand Kit (Qiagen). The PCR assay was carried out in 96-well PCR plates (RT2 Profiler PCR Array) using the FQD-96A system (Bio-Rad, Hercules, CA, United States) with the following parameters: an initial denaturing step at 95°C for 10 min, followed by 40 cycles of 95°C for 15 s and 60°C for 1 min. The global mRNA expression patterns of UPR molecules in foetal livers were analysed based on Student's *t*-test (Supplementary Table 2) and illustrated using K-means clustering in conjunction with a heatmap and volcano plot (http://www.qiagen.com.cn/shop/genes-and-pathways/data-analysis-center-overview-page/).

Routine qPCR techniques were used to validate the results of the PCR array, as well as to determine the differentially expressed genes in rat livers of different ages. Briefly, total RNA was extracted from hepatic tissues and reverse transcribed to cDNA as described above. Then, amplification was performed using a LightCycler 480 SYBR Green I Master (Roche Diagnostics, Basel, Switzerland) in 20 μL reaction solution using the primers described in Table 2. The amplicon size and specificity were confirmed by agarose gel electrophoresis (Figure **5B**). The relative mRNA levels were calculated using the 2^−ΔΔCt^ method after normalization against *Rplp1* as a housekeeping gene.

**Table 2:** Primers used for quantitative real-time reverse transcription PCR.

**Gene**	**Accession number**	**Sequence (5′−3′)**	**Product (bp)**	**Temp (°C)**
*Hspa4l*	NM_001106428	F: GCTTCATGGACGATCATTTG	113	59.08
		R: CCAGGTACCGCACCTTAACT		59.12
*Creb3l3*	NM_001012115	F: ATAGCGGTCCTTCTGCTGTC	77	59.46
		R: CTGTCGACTTTGTTGGCAGT		58.93
*Ern2*	NM_001108919	F: TGAGGAACAAGAAGCACCAC	99	58.85
		R: AGCGCTGTGTGAAGTATTGG		58.95
*Mapk10*	NM_012806	F: AAACTTAAAGCCAGCCAAGC	112	58.65
		R: ACCAAACGTTGATGTACGGA		58.9
*Hsf1*	NM_024393	F: GACATGAGCCTGCCTGACCT	126	58.92
		R: GTGCACCAGCTGCTTTCCTG		59.23
*Hsf2*	NM_031694	F: CTCCGCGTTCGGGAGTAGAA	69	58.74
		R: GGCACGTTGGAACTCTGCTT		58.24
*Ppar-a*	NM_013196	F: CGTGGTGCATTTGGGCGTAA	78	58.95
		R: TTCAGTCTTGGCTCGCCTCT		58.02
*Rplp1*	NM_001007604	F: CTGCATCTACTCCGCCCTCA	116	58.43
		R: ACAAGCCAGGCCAGAAAGGT		58.97

*Rplp1 was used as a housekeeping gene. F, forward primer, R, reverse primer*.

### Western blotting

Western blotting was used to detect the presence of UPR pathway proteins in rat livers at each time point, as previously described (10). Briefly, total protein was extracted using radio immunoprecipitation assay (RIPA) lysis buffer, and the concentration was determined using a bicinchoninic acid (BCA) protein assay kit. Equal amount of proteins were then separated by SDS-PAGE and transferred onto a polyvinylidene difluoride membrane. After transfer, the membrane was cut into several strips to detect different target proteins depending on the molecular weight. Hspa41, Hspa1l, c-Jun, and Mapk10 were run together with β-actin; so is Creb3l3 and Creb3l1; Atf2, Atf6, eIF2, and p-eIF2α; xbp1 and Grp78. The membrane was then blocked with 5% bovine serum albumin (BSA) and probed with the primary antibodies (Table 3), followed by probing with horseradish peroxidase-conjugated second antibodies for 2 h at room temperature. The antigen-antibody complexes were visualised using enhanced chemiluminescence (ECL) plus reagent (Millipore, Billerica, MA, United States). Gel-Pro analyser (Bio-Rad) was applied for analysis of the optical density of the protein bands with β-actin as the reference gene. The result was presented as the ratio of the targets to β-actin.

**Table 3:** Antibodies for western blotting and IHC.

**Target protein**	**Source**	**Manufacture**	**Number**	**WB dilution**	**MW (kDa)**
HSPA4L	Rabbit	Biorbyt	orb184242	1:500	94
HAPA1L	Rabbit	Proteintech	13970-1	1:2,000	90
XBP1	Rabbit	Enogene	E1A5110	1:1,000	s56/u24
ATF6	Rabbit	Proteintech	24169-1	1:500	100
MAPK10	Rabbit	Enogene	E1A5321	1:500	53
CREB3L1	Rabbit	Proteintech	11235-2	1:1,000	55
CREB3L3	Rabbit	Immunoway	YN1809	1:1,000	50
ATF2	Rabbit	Proteintech	14894-1	1:500	55
c-Jun	Rabbit	Proteintech	120024-2	1:500	55
GRP78	Rabbit	CST	3,177	1:1,000	78
eIF2α	Rabbit	CST	5,324	1:1,000	36
p-eIF2α	Rabbit	CST	3,398	1:500	38
β-actin	mouse	Proteintech	66009-1	1:5,000	42

*s56/u24, Xbp1s56kDa, Xbp1u24kDa; WB, western blot; CST, Cell Signalling Technology*.

### Statistical analysis

Statistical analysis of qPCR array data was performed using the SABiosciences web-based software for Standard RT^2^ PCR array analysis. The data for development index, food intake, blood glucose, and glycogen levels in hepatic tissues, as well as relative gene expression, were analysed using each rat as an experimental unit in the present study. All data were checked for normal distribution and homogeneity of variance using the Shapiro-Wilk test and the Levene Test, respectively. The data are presented as the mean ± standard error of the mean (SEM). Comparison of the means was performed using two-tailed Student's unpaired *t*-test with SPSS 17.0 (SPSS Inc., Chicago, IL, United States). A value of *p* < 0.05 was considered statistically significant.

## Results

### Weight and metabolic parameters

**Figure 2:**
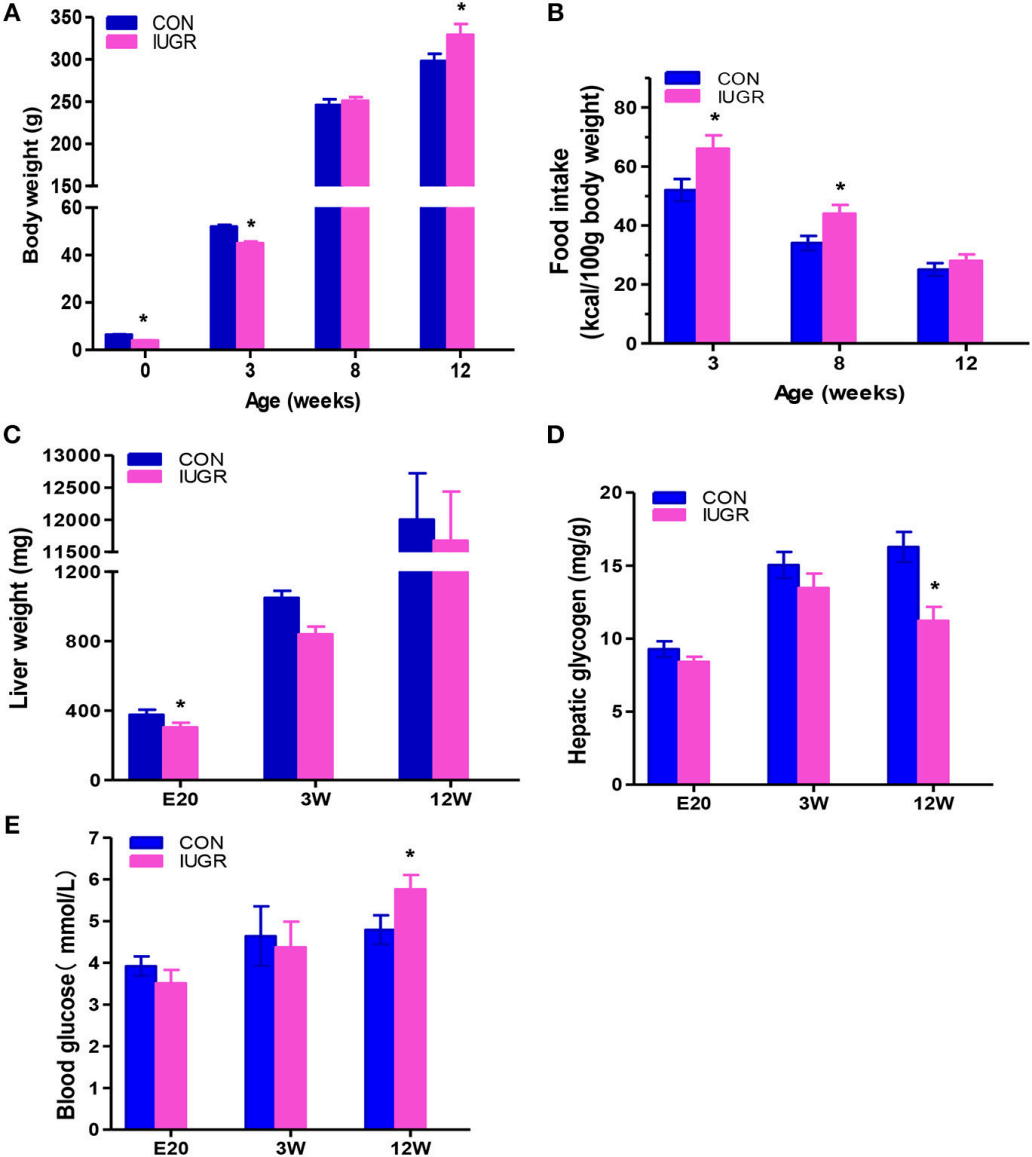
The effect of maternal protein restriction on offspring liver growth and metabolic profile. **(A)** Body weight (g) from birth to postnatal week 12. The data of different development stages were presented in the figure: Birth (control, *n* = 103; IUGR, *n* = 108), Weaning: postnatal week 3 (3 W; control, *n* = 34; IUGR, *n* = 31), Adolescent: 8 W (control, *n* = 24; IUGR, *n* = 22), and Adult:12 W (control, *n* = 19; IUGR, *n* = 18). **(B)** Food intake of control and IUGR rats. Food intake, expressed as a kcal/100 g body weight, was higher in the IUGR group compared to the control group at 3 and 12 week. **(C)** Hepatic weight at different time points was measured in IUGR and control rats. Hepatic weight was lower in the IUGR rats compared to the age matched control at E20. **(D)** Hepatic glycogen concentration at different time points was determined in IUGR and control rats. Hepatic glycogen amount in the adult IUGR rats was lower than that of adult control. **(E)** Fasting plasma glucose at different time points was examined in both groups. At 12 weeks, fasting plasma glucose increased in the IUGR group, exceeding the control group. Blue bars, control group; purple bars, IUGR group. Data are shown as the mean ± SEM. *p < 0.05 (*n* = 6 for both groups, unpaired Student's *t*-test); IUGR, intrauterine growth restriction. For all the research above, IUGR group referred to those offspring rats with the body weight of two standard deviations less than the mean body weight of the age-matched control on E20 or at birth.

There were no observed changes in average gestation period (21.5 days, ~21–22 days) or average litter size (undernourished: 10.3 ± 1.8 vs. control: 11.1 ± 1.8, *p* = 0.23) between the two groups. Maternal protein restriction resulted in foetal growth restriction, as evidenced by the reduced body weight (Figure 2A) and significantly higher incidence rate of IUGR (undernourished: 63.0%, 68/108, vs. control: 3.88%, 4/103, *p* < 0.001). Undernourished foetuses also showed a significant reduction in liver weight (*p* < 0.01). The postnatal body weight of IUGR animals was restored to normal after weaning; it then surpassed that of the control group at 12 W because of catch-up growth (Figure 2A), a phenotype similar to that observed in the human with IUGR (8). Food intake was higher in the IUGR group than in the control group at 3 and 12 W, which explained the catch-up growth during early stages in the IUGR group (Figure 2B). Consistent with other report (11), protein restriction resulted in a significant decrease in foetal liver weight. However, the weights of the restored livers were comparable to those of age-matched control animals at 3 and 12 W (Figure 2C). The blood glucose levels in the two groups at different time points are shown in Figure 2E. Fasting blood glucose was slightly decreased in IUGR foetuses, but remarkably higher in IUGR rats at 12 W than in control rats. There was no difference in hepatic glycogen levels at E20; however, the concentrations of glycogen were remarkably lower in IUGR offspring at 12 W (Figure 2D).

### Histological changes in liver glycogen storage

**Figure 3:**
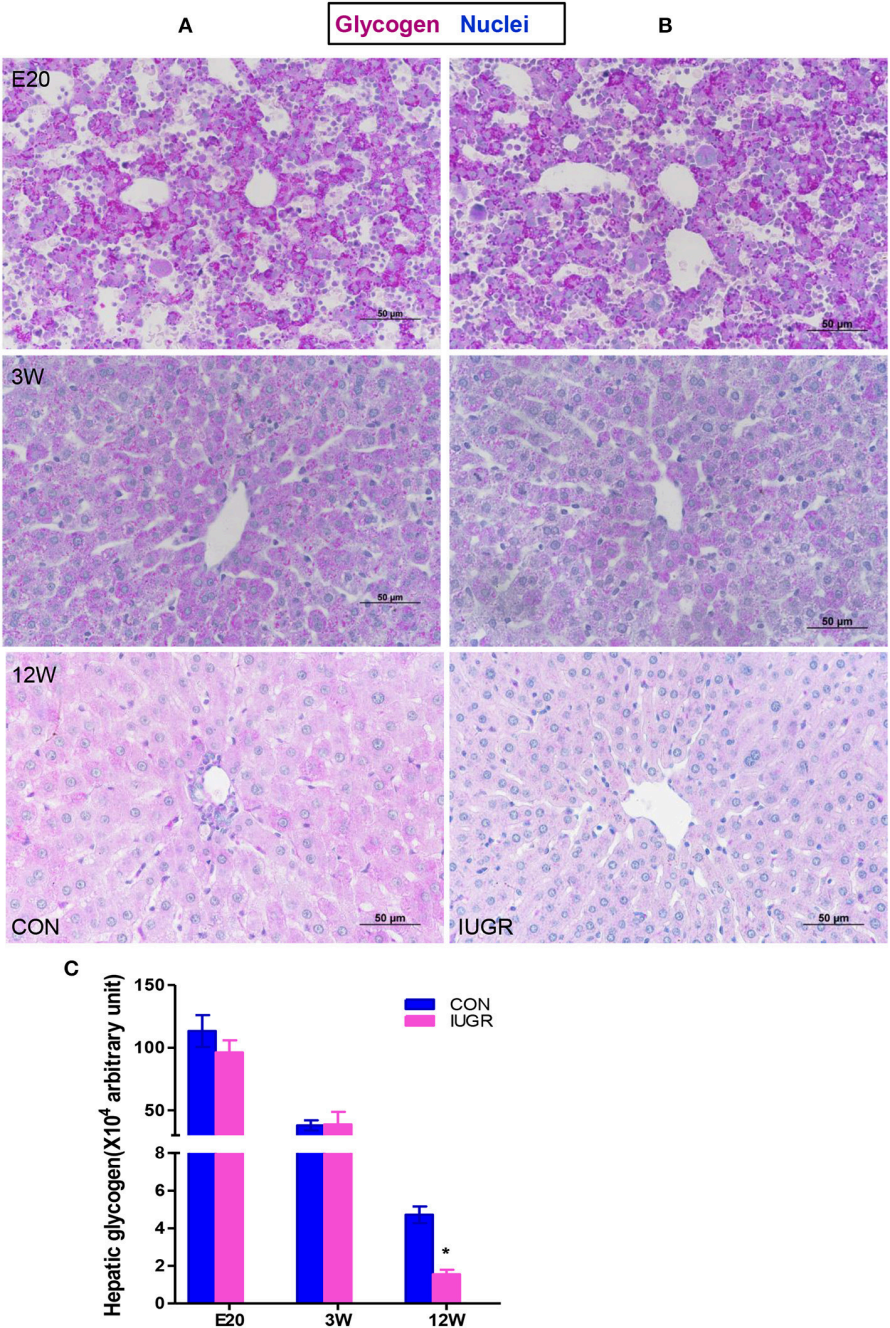
Hepatic glycogen contents in IUGR offspring. Representative hepatic sections showing periodic acid-Schiff (PAS) staining for glycogen (purple) from control **(A)** and IUGR **(B)** rats at E20, 3, and 12 W. **(C)** PAS staining was quantified in the control and IUGR groups [*n* = 6 per group; *p < 0.05 (unpaired Student's *t*-test) vs. age-matched controls]. Images were taken at 400× magnification using a light microscope; scale bar = 50 μm.

Hepatic glycogen is both a storage system and a ready source of blood glucose. To assess glycogen deposition in liver tissues, quantitative glycogen analysis was carried out using PAS staining (Figure 3). There were no significant differences in hepatic glycogen storage between the IUGR group and control group at the embryonic stage (E20) and adolescence (3 W), but IUGR animals exhibited lower PAS-positive staining at 12 W, consistent with the hyperglycaemia observed in adult IUGR rats.

### qPCR array analysis of UPR molecules

**Figure 4:**
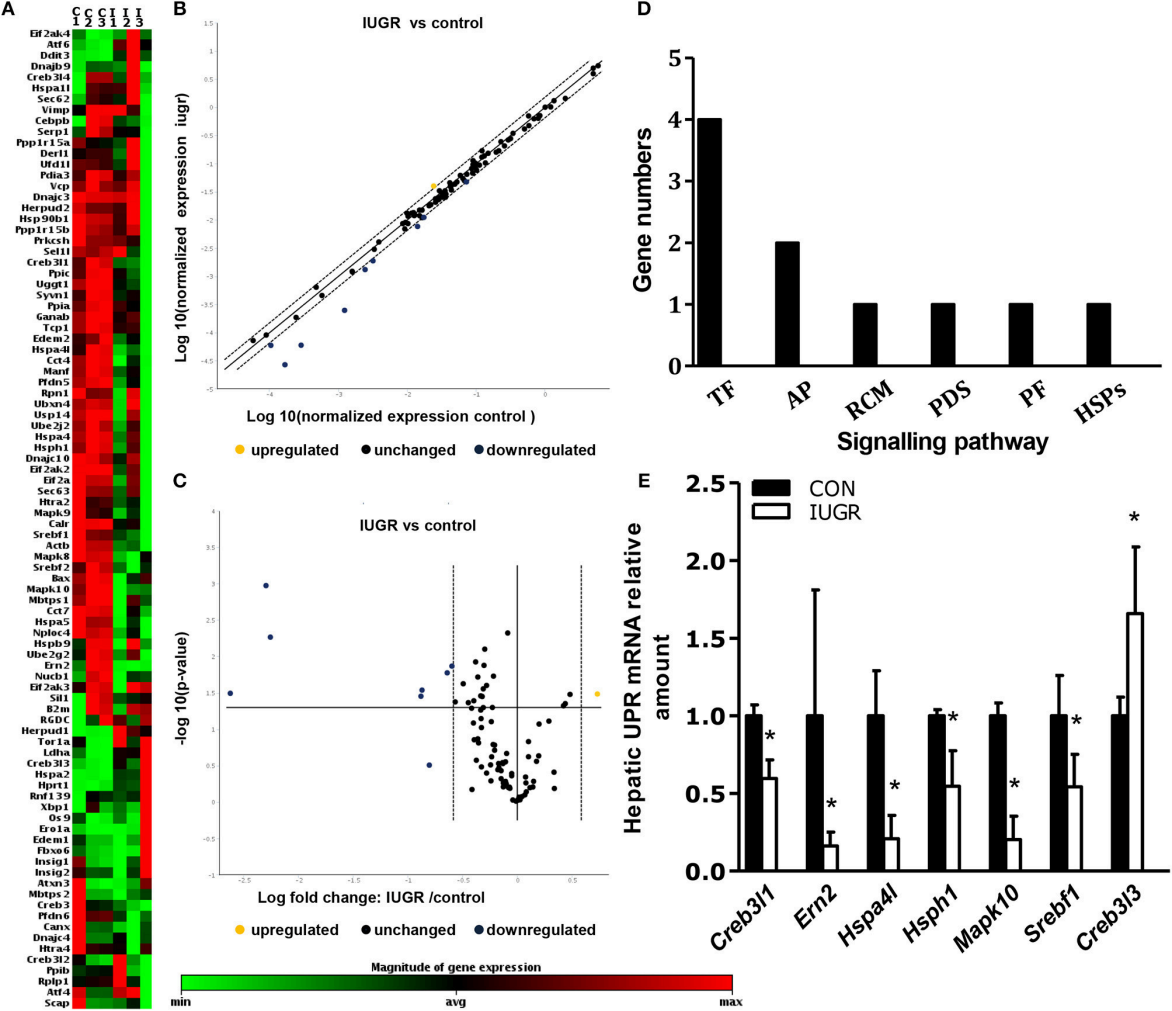
Determination of the expression of hepatic unfolded protein response (UPR)-related factors in IUGR foetuses by qPCR arrays. **(A)** K-means clustering representation of 84 mRNA profiles. The magnitude of the percentage is represented by a colour scale (top right) going from low (green) to high (red). **(B,C)** Scatter plot and volcano plot showing changes in mRNA expression between the two groups (*n* = 3). **(D)** Differentially expressed genes in six signalling pathways related to UPR in IUGR rats. RCM, regulation of cholesterol metabolism; ERAD, endoplasmic reticulum-associated degradation; TF, transcription factor; PF, protein folding; PDI, protein disulphide isomerization; HSP, heat-shock protein; Ap, apoptosis. **(E)** Differentially expressed genes between control and IUGR rats. The data are presented as the mean ±SEM. [*n* = 3 per group; *p < 0.05 vs. control (unpaired Student's *t*-test].

We identified the significance and magnitude of the changes in the expression of UPR-related genes between the two groups using K-means clustering heatmaps, scatter plots, and volcano plots (Figures 4A–C). A vast majority of the genes showed no differences in expression. Of the eighty-four detected UPR-related genes (Supplementary Table 2), the mRNA levels of seven genes in IUGR foetuses changed significantly (fold-change > 1.5; *p* < 0.05) These genes encoded proteins involved in critical UPR events, and corresponded to six categories of signal pathways (Figure 4D). The mRNA levels of Hspa4l, endoplasmic reticulum to nucleus signalling 2 (Ern2), and Mapk10 were lower in IUGR foetal livers (E20), whereas Creb3l3 mRNA levels were increased compared to control foetuses (Figure 4E).

### Expression of hepatic UPR factors in IUGR foetuses

**Figure 5:**
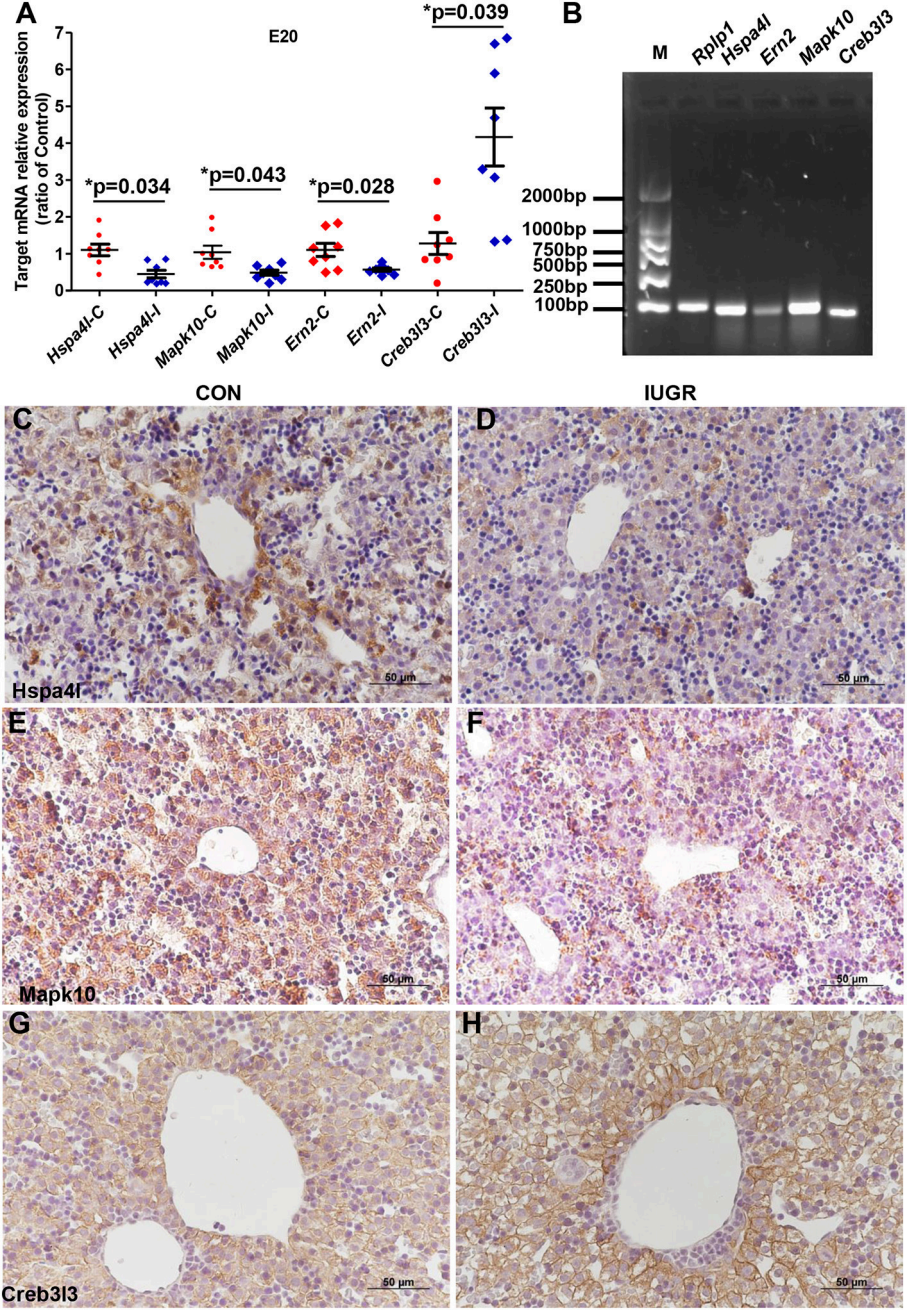
Determination of differentially expressed hepatic UPR-related genes in IUGR foetal livers. **(A)** qPCR analysis showing the mRNA expression of hepatic UPR-related genes in foetal rats. Data are expressed as mean ± SEM. *p < 0.05 vs. age-matched controls (*n* = 8). **(B)** Representative agarose gel electrophoresis images of PCR products confirmed the amplicon size and specificity. **(C–H)** Histological expression of UPR factors in the fetal hepatics. Representative photomicrographs of IHC staining for heat shock protein a4l (Hspa4l) **(C,D)**, mitogen-activated protein kinase 10 (Mapk10) **(E,F)** and Creb3l3 **(G,H)** in the liver sections from control and IUGR rats (Magnification: 400×; scale bar = 50 μm). At E20, hepatic Hspa4l and Mapk10 protein levels was decreased in IUGR foetuses compared to age-matched controls, while Creb3l3 increased in fetal livers.

In order to confirm the results of the PCR array, qPCR was used to quantify the expression of Hspa4l, Mapk10, Ern2, and Creb3l3 using an expanded sample size (*n* = 8); Rplp1 was used as the housekeeping gene, as it showed the most stable expression of the five internal reference genes from the PCR array. The genes were chosen based on the following criteria: fold-change > 2.0 and *p* ≤ 0.05 between IUGR and control samples. Maternal protein restriction and subsequent IUGR significantly decreased hepatic Hspa4l, Mapk10, and Ern2 mRNA levels and increased Creb3l3 expression compared to the control, confirming the results of the array data (Figure 5A).

**Figure 6:**
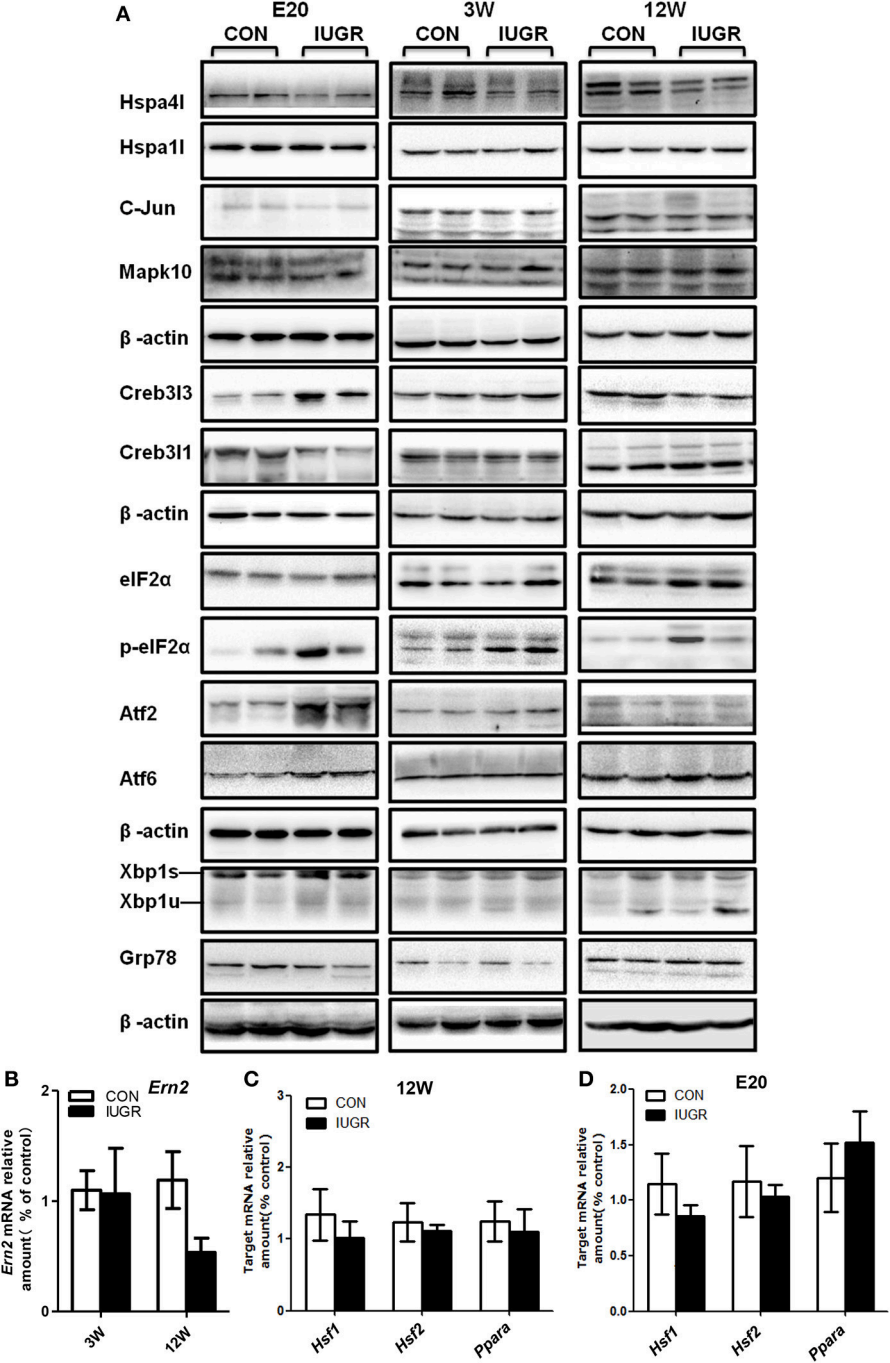
Determination of the protein levels of hepatic UPR-related factors and transcription factors at different ages by western blots. **(A)** Representative photographs of immunoblotting analysis for UPR-related factors at E20, 3, and 12 W. Western blot analysis was conducted in 5–8 animals in each group, and only representative blots are shown. **(B)** Determination of the mRNA levels of *Ern2* at different ages using qPCR. **(C,D)** Determination of the mRNA levels of transcription factors *Ppar-*α, *Hsf1*, and *Hsf2* at different ages using qPCR. Data are expressed as the mean ± SEM. (*n* = 6–8, unpaired Student's *t*-test). IUGR group referred to those offspring rats with the body weight of two standard deviations less than the mean body weight of the age-matched control on E20 or at birth.

Furthermore, the protein levels of differentially expressed UPR components in foetuses were quantified by IHC and/or western blotting, with β-actin as the reference protein. Major differences were found in the production of several UPR-related proteins. At E20, the IHC staining intensity of Hspa4l (Figures 5C,D) and Mapk10 (Figures 5E,F) was decreased in embryos with IUGR compared to age-matched controls, while the intensity of Creb3l3 staining increased in IUGR foetuses (Figures 5G,H). The results of IHC were consistent with the mRNA results. Immunoblotting also showed that the protein levels of Hspa4l, Creb3l1, and Mapk10 decreased significantly in IUGR foetuses (E20) compared to age-matched controls, while the levels of Creb3l3 protein increased remarkably (Figure 6A and Table 4), consistent with the mRNA data. The statistical data was presented in Table 4. Interestingly, there was no difference in Hspa1l protein activity between the two groups at E20 (Figure 6A), although its mRNA levels were decreased in the IUGR group. This indicated that in addition to transcriptional regulation, the expression of UPR genes can be regulated at the translational or post-translational levels. We further investigated the protein level and activity of several key factors in the UPR pathway by western blotting. GRP78 is a major chaperone that controls the activation of ER-transmembrane signaling mechanisms. Hepatic Grp78 protein levels showed a tendency of mild decrease (−32%, *p* = 0.12) in IUGR groups at E20. However, results for Grp78 protein at 3 and 12 W did not show any differences between two groups (Figure 6A).Spliced X-box binding protein 1 (XBP1s). an active transcriptional factor which induces ER metabolic components, showed a tendency to be higher in IUGR foetal livers (+95%, *p* = 0.066), while restored to normal at 3 and 12 W (Figure 6A and Table 4). The protein levels of Atf6 and Atf2, two important UPR genes, were significantly increased in IUGR foetal livers (Figure 6A and Table 4); however, the mRNA levels of these genes were similar between two groups detected by PCR Array. No significant changes were observed in the protein levels of c-Jun or eIF2α at E20, while the phosphorylation of eIF2α increased significantly in the IUGR group compared to the control group (Figure 6A and Table 4). These results indicated that some changes may have occurred at the translational or post-translational levels, rather than at the transcriptional level.

**Table 4:** Western blots results showing the change of hepatic UPR factors at different development stages.

**Protein**	**IUGR/CON (E20)**	**IUGR/CON (3 W)**	**IUGR/CON (12 W)**
Hspa4l	0.128 ± 0.013/0.20 ± 0.015↓^*^	0.405 ± 0.073/1.164 ± 0.309 ↓^*^	0.268 ± 0.095/0.763 ± 0.148 ↓^*^
Hspa1l	NC	NC	NC
Creb3l3	1.008 ± 0.311/0.254 ± 0.051↑^*^	1.666 ± 0.384/0.925 ± 0.2 ↑(*p* = 0.118)	NC
Creb3l1	0.077 ± 0.004/0.242 ± 0.054↓^*^	NC	NC
Mapk10	0.377 ± 0.044/0.675 ± 0.084↓^*^	NC	NC
eIF2α	NC	NC	NC
p-eIF2α	0.553 ± 0.158/0.155 ± 0.055↑^*^	0.126 ± 0.036/0.044 ± 0.0070 ↑ (*p* = 0.052)	1.382 ± 0.471/0.805 ± 0.425 ↑(*p* = 0.14)
c-Jun	NC	NC	NC
Atf2	0.229 ± 0.0646/0.034 ± 0.00↑^*^	NC	NC
Atf6	0.173 ± 0.029/0.084 ± 0.011↑^*^	NC	1.823 ± 0.415/0.591 ± 0.087↑^*^
Xbp1s	0.27 ± 0.13/0.14 ± 0.09 ↑ (*p* = 0.066)	NC	NC
Xbp1u	NC	NC	NC
Grp78	NC	NC	NC

*Data are expressed as mean ± SEM*.

* *p < 0.05 vs. age-matched controls (n = 5–8)*.

*NC, no change*. ↑/↓ *means increased/decreased respectively, compared with age-matched controls*.

### The expression of UPR genes at different ages

In order to explore whether the changes in hepatic gene expression were transient or permanent, we continued to investigate the hepatic UPR signalling pathway in adolescent (3 W) and adult (12 W) rats. After birth, the expression of most UPR genes including Creb3l1, Creb3l3, and Mapk10 returned to normal at 3 or 12 W, which indicated a temporal window of vulnerability. However, the dysregulation of Hspa4l was maintained until 12 W (Figure 6A and Table 4). The phosphorylation levels of eIF2α also remained higher until 3 W (Figure 6A and Table 4). The mRNA expression of Ern2 was determined at 12 W by qPCR; as there is no antibody specific to its rat homologue, the protein level of this factor could not be directly determined. Our results indicated that Ern2 expression was restored to normal levels at 3 and 12 W (Figure 6B).

To screen for any upstream signals that may regulate the expression of Hspa4l and Creb3l3, qPCR analysis of the transcription factors heat-shock factor 1 (HSF1), HSF2, and peroxisome proliferator-activated receptor-α (Ppar-α) were performed (Figures 6C,D). The results showed that the mRNA levels of Hsf1 and Hsf2 were unchanged, and did not correlate with the expression of Hspa4l. The mRNA levels of Ppar-α were slightly increased at E20, but were restored to normal levels at 12 W, and did not correlate with the expression of Creb3l3. These results indicate that other transcription factors responsible for the deregulation of HSP and Creb3l3 in IUGR hepatic tissues must be investigated.

## Discussion

Numerous epidemiological investigations have demonstrated a significant association between birth weight and postnatal hepatic function (2). Consistent with other reports, using a well-characterized IUGR rat model, our previous studies (10, 27) have demonstrated that IUGR predisposes offspring to hyperglycaemia and hepatic mild steatosis later in life, which indicated that IUGR increase the susceptibility to metabolic disorders. Experimental models have demonstrated that maternal programming has great influence on ER stress, which plays a vital role in the pathogenesis of NAFLD (23, 28). Stefano reported recently that IUGR induced by uteroplacenta insufficiency led to activation of hepatic UPR in offspring rats, confirmed by upregulated expression of XBP1 and PERK, which indicated that UPR signalling may play a role in the metabolic risk (29). Using pathway-specific PCR arrays, we examined the expression of eighty-nine genes associated with UPR in the livers of IUGR foetuses. Our results indicate that of those eighty-nine ER stress genes, the expression of seven genes in the livers of IUGR rats were deregulated at E20; some returned to normal levels at 3 and 12 W. The deregulation of Hspa4l was maintained from foetal to adult stages. In contrast to the reports by Riddle et al. (25), we did not observe any changes in the hepatic mRNA or protein levels of PERK, ATF4, or GRP78 in IUGR foetuses, possibly because we analysed different tissues or from different rat models (uteroplacental insufficiency-induced) and different developmental stages in our study. These results indicated that IUGR caused by different aetiologies might lead to different changes in the liver, and one type of rat model cannot completely simulate the condition of human diseases. In addition, maternal low protein diet has an adverse effect on hepatic function, owing to an irreversible alteration during embryo development. However, the fact that the isocaloric low protein diet is slightly higher in carbohydrates and fat than the control as well as lower in protein may also have an independent impact on glucose and lipid metabolism.

The ER is the location of biosynthesis for sterols, lipids, and membrane and secretory proteins, and has strict quality control mechanisms that allow the secretion of correctly folded proteins into the cytoplasm. In hepatocytes, over 90% of translated polypeptides are directed into the ER lumen for folding and assembly (30), and the protein folding system plays important physiological roles in the ER (31, 32). Differentially expressed genes in IUGR foetuses were associated with six different categories of ER stress factors. HSPs act as molecular chaperones by assisting the folding of nascent and misfolded proteins, preventing their aggregation (33). HSPA4L belongs to the HSP110 protein family, which consists of four genes: *Hspa4l, Hspa4, Hsph1*, and *Grp175* (34, 35). The HSP110 family act as cochaperones of HSP70 chaperones (36). In IUGR livers, the decreased expression of Hspa4l at both the mRNA and protein levels could lead to a decrease in protein folding ability. Unfolded or misfolded protein accumulation in the ER can induce ER stress and activate signalling pathways including PERK, ATF6, and IRE1 (15). A number of transcription factors were downregulated, indicating that the aberrant regulation of UPR factors at the transcriptional level may play an important role in the pathogenesis of hepatic malfunction caused by IUGR. Expression of the transcription factor *Ern2* decreased at E20. Ern2 is required for the transcriptional regulation of genes involved in lipogenesis metabolism, and it plays a vital role in regulating microsomal triglyceride transfer protein (MTP) and chylomicron production. The upregulation of Ern2 may be beneficial in avoiding diet-induced hyperlipidaemia (37); therefore, the downregulation of hepatic Ern2 in IUGR foetuses may be associated with higher postnatal cholesterol levels, consistent with our previous report. CREB3L3 is highly expressed during hepatogenesis. In addition to its role in the mobilization of the acute phase response to ER stress by the transcriptional regulation of acute phase response genes such as those encoding C-reactive protein and amyloid P-component, CREB3L3 is necessary for the transcriptional activation of genes involved in lipogenesis, fatty acid and cholesterol metabolism, and glucose metabolism (38). Several other important genes, such as those encoding phosphoenolpyruvate carboxykinase (PEPCK-C) and glucose 6 phosphatase (G6Pase), are also direct targets of Creb3l3.

The overexpression or constitutive activation of Creb3l3 activates the transcription of PEPCK-C or G6Pase; the knockdown of Creb3l3 in the liver significantly reduces blood glucose levels (39). The hepatic mRNA levels of gluconeogenic genes were reduced proportionally after the depletion of Creb3l3 (39). Our previous study showed a marked increase in the expression of PEPCK and G6Pase in the livers of IUGR rats from E20 to 12W (27). It is possible that the upregulation of Creb3l3 induces the overexpression of glucogenesis enzymes that may alter hepatic glycogen synthesis and breakdown. Thus, chromatin immunoprecipitation (ChIP) and qPCR analyses would be useful in investigating the contribution of Creb3l3 at gluconeogenic gene promoters to the regulation of gene expression in IUGR animals.

In addition to transcriptional regulation, the expression of UPR genes can be regulated at the translational or post-translational levels. For example, although the *Atf6* and *Atf2* mRNA levels were similar between the IUGR and control groups, both proteins were significantly increased in the IUGR group, especially Atf6, which was upregulated until 12 W. Atf6 is a transcription factor with a major role in the regulation of ER quality control proteins, and plays dual protective and pathologic roles in fatty liver disease due to ER stress (14). Interesting, Oben et al. reported that maternal obesity modified the hepatic UPR rhythmicity with Atf6 activation during the day, which was aggravated by the double obesogenic hit (combination of intrauterine and postpartum obesogenic diets) (23).Both intrauterine nutrition deficiency and overnutrition trigged upregulated ATF6, indicating that ATF6 play an important role in the pathogenesis of developmental origins of NAFLD. The hepatic metabolic disorder in IUGR model was mild. We speculated that the phenotype in IUGR offspring would be aggravated if fed with high-fat diet postpartum, which would be more favourable for investigating the changes of UPR pathway, and the association with the developmental programming of NAFLD. The overexpression of Atf6 also inhibited transcription factor sterol regulatory binding protein (SREBP)-mediated transcription, and the expression of its target genes (40). SREBP is the master regulator of triglyceride and cholesterol synthesis in hepatocytes, and is essential for obesity- and alcohol-induced steatosis (41). Besides regulating lipid metabolism, ATF6 also regulate hepatic glucose output bymediating CREB regulated transcription coactivator 2 (CRTC2) recruitment to ER stress inducible promoters, and disrupting the CREB:CRTC2 interaction and thereby inhibiting CRTC2 occupancy over gluconeogenic genes (42). We speculated that increased protein level of hepatic Atf6 in IUGR offspring may disrupt the function ofcritical factors that regulated glucose and lipid metabolism, subsequently promoting lipid accumulation and fatty liver; this requires further investigation.

In summary, this study used an *in vivo* model to analyse the changes in gene expression in the livers of animals with IUGR. The results indicated that an adverse intrauterine environment affected the lifelong expression of key factors involved in the UPR pathway, which may alter cholesterol and glucose metabolism. Given the plasticity of the liver during foetal and neonatal stages, understanding the impact of IUGR on the liver may be potentially helpful for the development of early life dietary and drug treatment strategies to reduce the incidence of metabolic syndrome in adulthood.

## Author contributions

XL conceived, designed and performed q-PCR array of the experiments, and also wrote the manuscript. LG managed the animals and collected samples. JW performed the pathology experiments and analyzed the data. YJ and CL helped review the manuscript. All authors read and approved the submitted version.

### Conflict of interest statement

The authors declare that the research was conducted in the absence of any commercial or financial relationships that could be construed as a potential conflict of interest.

## Abbreviations

IUGR: intrauterine growth restriction
NAFLD: nonalcoholic fatty liver disease
HSPA4L: Heat-shock 70-kDa protein 4L
FBG: fasting blood glucose
MAPK10: Mitogen-activated protein kinase 10
CREB3L: Cyclic AMP-responsive element-binding protein 3-like protein
ERN2: endoplasmic reticulum to nucleus signalling 2
EIF2: eukaryotic translation initiation factor 2
PEPCK: phosphoenolpyruvate carboxykinase
G6Pase: glucose-6-phosphatase
UPR: Unfolded protein response
XBP1: X-box binding protein 1
ER: Endoplasmic reticulum
CRTC2: CREB regulated transcription coactivator 2
GRP: Glucose-regulated protein
IRE1: Inositol requiring 1
ATF6: Activating transcription factor 6
PERK: Protein kinase R-like endoplasmic reticulum kinase
ERSR: ER stress response
ERAD: ER-associated protein degradation
IHC: immunohistochemically
PAS: periodic acid shift
HPS: heat shock protein
HSF: heat shock transcription factor
PPARα: Peroxisome proliferator-activated receptor alpha.
