# Supplementary material for: Maternal Protein Restriction Induces Alterations in Hepatic Unfolded Protein Response-Related Molecules in Adult Rat Offspring
Source: Front Endocrinol (Lausanne). 2018 Nov 20;9:676. doi: 10.3389/fendo.2018.00676 (PMC6262354; doi:10.3389/fendo.2018.00676)
Supplement: Supplementary file 1 [file Table_1.DOCX]

**Supple Table 1:** Q-PCR array gene table. A total of 96 genes, including 84 ER stress-related factors, five housekeeping genes (H01-05), and seven quality control genes (H06-12), were detected in q-PCR arrays.

| Position | Unigene | Refseq | Symbol | Description |
| --- | --- | --- | --- | --- |
| A01 | Rn.107561 | XM_341644 | AMFR | Autocrine motility factor receptor |
| A02 | Rn.161941 | NM_001108183 | ARMET | Arginine rich, mutated in early-stage tumors |
| A03 | Rn.2423 | NM_024403 | ATF4 | Activating transcription factor 4 (tax-responsive enhancer element B67) |
| A04 | Rn.222130 | NM_001107196 | ATF6 | Activating transcription factor 6 |
| A05 | Rn.18179 | NM_001002809 | ATF6B | Activating transcription factor 6 beta |
| A06 | Rn.42932 | NM_021702 | ATXN3 | Ataxin 3 |
| A07 | Rn.10668 | NM_017059 | BAX | BCL2-associated X protein |
| A08 | Rn.974 | NM_022399 | CALR | Calreticulin |
| A09 | Rn.1762 | NM_172008.2 | CANX | Calnexin |
| A10 | Rn.97889 | NM_182814.2 | CCT4 | Chaperonin containing TCP1, subunit 4 (delta) |
| A11 | Rn.62267 | NM_001106603.1 | CCT7 | Chaperonin containing TCP1, subunit 7 (eta) |
| A12 | Rn.6479 | NM_024125.4 | CEBPB | CCAAT/enhancer binding protein (C/EBP), beta |
| B01 | Rn.104043 | NM_001013092.1 | CREB3 | CAMP responsive element binding protein 3 |
| B02 | Rn.20059 | NM_001012115.1 | CREB3L3 | CAMP responsive element binding protein 3-like 3 |
| B03 | Rn.11183 | NM_001109986 | DDIT3 | DNA-damage-inducible transcript 3 |
| B04 | Rn.110990 | NM_001014202.1 | DERL1 | Der1-like domain family, member 1 |
| B05 | Rn.11209 | NM_031627 | CHOP | Rattus norvegicus nuclear receptor subfamily 1, group H, member 3 |
| B06 | Rn.40780 | NM_001109541 | DNAJB2 | DnaJ (Hsp40) homolog, subfamily B, member 2 |
| B07 | Rn.29778 | NM_012699 | DNAJB9 | DnaJ (Hsp40) homolog, subfamily B, member 9 |
| B08 | Rn.8642 | NM_001106486 | DNAJC10 | DnaJ (Hsp40) homolog, subfamily C, member 10 |
| B09 | Rn.162234 | NM_022232 | DNAJC3 | DnaJ (Hsp40) homolog, subfamily C, member 3 |
| B10 | Rn.91398 | NM_001013196 | DNAJC4 | DnaJ (Hsp40) homolog, subfamily C, member 4 |
| B11 | Rn.107459 | NM_001033909 | Elf2 | E74-like factor 2 |
| B12 | Rn.81078 | NM_130422 | Casp12 | Caspase 12 |
| C01 | Rn.198593 | NM_001109339 | eIF2A | Eukaryotic translation initiation factor 2A, 65 kDa |
| C02 | Rn.24897 | NM_031599 | EIF2AK3 | Eukaryotic translation initiation factor 2-alpha kinase 3 |
| C03 | Rn.19198 | NM_001037208 | CRELD2 | cysteine-rich with EGF-like domains 2 |
| C04 | Rn.218563 | XM_344959.3 | ERN2 | Endoplasmic reticulum to nucleus signaling 2 |
| C05 | Rn.64648 | NM_138528 | ERO1L | ERO1-like (S. cerevisiae) |
| C06 | Rn.22325 | NM_144755 | TRB3 | Tribbles homolog 3 |
| C07 | Rn.2459 | NM_001008317 | ERP44 | Thioredoxin domain containing 4 (endoplasmic reticulum) |
| C08 | Rn.57325 | NM_138917 | FBXO6 | F-box protein 6 |
| C09 | Rn.99241 | NM_001106334 | GANAB | Glucosidase, alpha; neutral AB |
| C10 | Rn.23744 | NM_001145840 | GANC | Glucosidase, alpha; neutral C |
| C11 | Rn.4028 | NM_053523 | HERPUD1 | Homocysteine-inducible, endoplasmic reticulum stress-inducible, ubiquitin-like domain member 1 |
| C12 | Rn.1950 | NM_212504 | HSPA1B | Heat shock 70 kDa protein 1B |
| D01 | Rn.187184 | NM_212546 | HSPA1L | heat shock protein 1-like |
| D02 | Rn.211303 | NM_021863 | HSPA2 | Heat shock protein 2 |
| D03 | Rn.163092 | NM_153629 | HSPA4 | Heat shock protein 4 |
| D04 | Rn.144829 | NM_001106428 | HSPA4L | Heat shock protein 4-like |
| D05 | Rn.11088 | NM_013083 | HSPA5 | Heat shock 70 kDa protein 5 (glucose-regulated protein, 78 kDa) |
| D06 | Rn.37805 | NM_001011901 | HSPH1 | Heat shock 105 kDa/110 kDa protein 1 |
| D07 | Rn.107325 | NM_001106599 | HTRA2 | HtrA serine peptidase 2 |
| D08 | Rn.163330 | NM_001107321 | HTRA4 | HtrA serine peptidase 4 |
| D09 | Rn.772 | NM_022392 | INSIG1 | Insulin-induced gene 1 |
| D10 | Rn.16736 | NM_178091 | INSIG2 | Insulin-induced gene 2 |
| D11 | Rn.9911 | NM_012806 | MAPK10 | Mitogen-activated protein kinase 10 |
| D12 | Rn.4090 | XM_001056513 | MAPK8 | Mitogen-activated protein kinase 8 |
| E01 | Rn.9910 | NM_017322 | MAPK9 | Mitogen-activated protein kinase 9 |
| E02 | Rn.2362 | NM_053569 | MBTPS1 | Membrane-bound transcription factor peptidase, site 1 |
| E03 | Rn.212224 | NM_001035007 | MBTPS2 | Membrane-bound transcription factor peptidase, site 2 |
| E04 | Rn.144645 | NM_080577 | NPLOC4 | Nuclear protein localization 4 homolog (S. cerevisiae) |
| E05 | Rn.1492 | NM_053463 | NUCB1 | Nucleobindin 1 |
| E06 | Rn.1579 | NM_001007265 | OS9 | Osteosarcoma amplified 9, endoplasmic reticulum associated protein |
| E07 | Rn.11527 | NM_017319 | PDIA3 | Protein disulfide isomerase family A, member 3 |
| E08 | Rn.7627 | NM_001109476 | PFDN2 | Prefoldin subunit 2 |
| E09 | Rn.3401 | NM_001106794 | PFDN5 | Prefoldin subunit 5 |
| E10 | Rn.1463 | NM_017101 | PPIA | Peptidylprolyl isomerase A (cyclophilin A) |
| E11 | Rn.2232 | NM_133546 | PPP1R15A | Protein phosphatase 1, regulatory (inhibitor) subunit 15A |
| E12 | Rn.104417 | NM_001106806 | PRKCSH | Protein kinase C substrate 80K-H |
| F01 | Rn.209127 | NM_001127545 | RNF139 | Ring finger protein 139 |
| F02 | Rn.209127 | NM_006913 | RNF5 | Ring finger protein 5 |
| F03 | Rn.4224 | NM_013067 | RPN1 | Ribophorin I |
| F04 | Rn.99548 | NM_001100966 | SCAP | SREBF chaperone |
| F05 | Rn.98327 | NM_001034129 | SEC62 | SEC62 homolog (S. cerevisiae) |
| F06 | Rn.24233 | NM_001107637 | SEC63 | SEC63 homolog (S. cerevisiae) |
| F07 | Rn.20802 | NM_177933 | SEL1L | Sel-1 suppressor of lin-12-like (C. elegans) |
| F08 | Rn.4197 | NM_173120 | SELS | Selenoprotein S |
| F09 | Rn.2119 | NM_030835 | SERP1 | Stress-associated endoplasmic reticulum protein 1 |
| F10 | Rn.103851 | NM_199376 | SIL1 | SIL1 homolog, endoplasmic reticulum chaperone (S. cerevisiae) |
| F11 | Rn.221929 | XM_001075680 | SREBF1 | Sterol regulatory element binding transcription factor 1 |
| F12 | Rn.41063 | NM_001033694 | SREBF2 | Sterol regulatory element binding transcription factor 2 |
| G01 | Rn.162486 | NM_001100739 | SYVN1 | Synovial apoptosis inhibitor 1, synoviolin |
| G02 | Rn.7102 | NM_012670 | TCP1 | T-complex 1 |
| G03 | Rn.20041 | NM_153303 | TOR1A | Torsin family 1, member A (torsin A) |
| G04 | Rn.139603 | NM_001106380 | UBE2G2 | Ubiquitin-conjugating enzyme E2G 2 (UBC7 homolog, yeast) |
| G05 | Rn.106299 | NM_001007655 | UBE2J2 | Ubiquitin-conjugating enzyme E2, J2 (UBC6 homolog, yeast) |
| G06 | Rn.2022 | NM_001012025 | UBXN4 | UBX domain protein 4 |
| G07 | Rn.11946 | NM_053418 | UFD1L | Ubiquitin fusion degradation 1-like (yeast) |
| G08 | Rn.162227 | NM_133596 | UGCGL1 | UDP-glucose ceramide glucosyltransferase-like 1 |
| G09 | Rn.107678 | NM_019381 | BI-1 | Transmembrane BAX inhibitor motif containing 6 |
| G10 | Rn.11790 | NM_001008301 | USP14 | Ubiquitin-specific peptidase 14 (tRNA-guanine transglycosylase) |
| G11 | Rn.98891 | NM_053864 | VCP | Valosin-containing protein |
| G12 | Rn.101044 | NM_001004210 | XBP1 | X-box binding protein 1 |
| H01 | Rn.973 | NM_001007604 | Rplp1 | Ribosomal protein, large, P1 |
| H02 | Rn.47 | NM_012583 | Hprt | Hypoxanthine guanine phosphoribosyl transferase |
| H03 | Rn.92211 | NM_173340 | Rpl13a | Ribosomal protein L13A |
| H04 | Rn.107896 | NM_017025 | Ldha | Lactate dehydrogenase A |
| H05 | Rn.94978 | NM_031144 | Actb | Actin, beta |
| H06 | N/A | U26919 | RGDC | Rat genomic DNA contamination |
| H07 | N/A | SA_00104 | RTC | Reverse Transcription Control |
| H08 | N/A | SA_00104 | RTC | Reverse transcription control |
| H09 | N/A | SA_00104 | RTC | Reverse transcription control |
| H10 | N/A | SA_00103 | PPC | Positive PCR control |
| H11 | N/A | SA_00103 | PPC | Positive PCR control |
| H12 | N/A | SA_00103 | PPC | Positive PCR control |

**Supplement table 2** Relative expression of the 89 genes by PCR Arrays

| Gene Symbol | AVG DeltaCt | | 2^(-DeltaCt) | | Fold Difference（I/C） | T-TEST  (p value) | Up- or Down- Regulation（I/C） |
| --- | --- | --- | --- | --- | --- | --- | --- |
|  | **IUGR** | **Control** | **IUGR** | **Control** |  |  |  |
| Atf4 | 2.71 | 2.75 | 0.152 | 0.148 | 1.03 | 0.841 | 1.03 |
| Atf6 | 6.24 | 6.44 | 0.013 | 0.012 | 1.15 | 0.085 | 1.15 |
| Atxn3 | 6.06 | 6.06 | 0.015 | 0.015 | 1 | 0.943 | 1 |
| Bax | 5.32 | 5.1 | 0.025 | 0.029 | 0.86 | 0.019 | -1.16 |
| Calr | -0.52 | -0.97 | 1.437 | 1.963 | 0.73 | 0.043 | -1.37 |
| Canx | 1.85 | 1.67 | 0.277 | 0.314 | 0.88 | 0.359 | -1.13 |
| Cct4 | 1.08 | 0.78 | 0.472 | 0.581 | 0.81 | 0.008 | -1.23 |
| Cct7 | 1.28 | 1 | 0.412 | 0.501 | 0.82 | 0.025 | -1.22 |
| Cebpb | 12.39 | 12.02 | 0.000 | 0.000 | 0.77 | 0.267 | -1.3 |
| Creb3 | 4.53 | 4.36 | 0.043 | 0.049 | 0.89 | 0.295 | -1.13 |
| Creb3l1 | 9.05 | 8.31 | 0.002 | 0.003 | 0.6 | 0.005 | -1.68 |
| Creb3l2 | 7.94 | 8.02 | 0.004 | 0.004 | 1.06 | 0.723 | 1.06 |
| Creb3l3 | 4.65 | 5.38 | 0.040 | 0.024 | 1.66 | 0.033 | 1.66 |
| Creb3l4 | 13.43 | 13.46 | 0.000 | 0.000 | 1.02 | 0.929 | 1.02 |
| Ddit3 | 6.25 | 6.67 | 0.013 | 0.010 | 1.34 | 0.048 | 1.34 |
| Derl1 | 5.06 | 4.98 | 0.030 | 0.032 | 0.94 | 0.632 | -1.06 |
| Dnajb9 | 4.45 | 4.6 | 0.046 | 0.041 | 1.11 | 0.519 | 1.11 |
| Dnajc10 | 7.17 | 6.77 | 0.007 | 0.009 | 0.76 | 0.139 | -1.32 |
| Dnajc3 | 1.93 | 1.78 | 0.262 | 0.291 | 0.9 | 0.477 | -1.11 |
| Dnajc4 | 8.37 | 8.24 | 0.003 | 0.003 | 0.91 | 0.619 | -1.09 |
| Tor1a | 4.93 | 5.1 | 0.033 | 0.029 | 1.13 | 0.276 | 1.13 |
| Edem1 | 2.94 | 3.05 | 0.130 | 0.120 | 1.08 | 0.635 | 1.08 |
| Edem2 | 4.93 | 4.83 | 0.033 | 0.035 | 0.93 | 0.215 | -1.07 |
| Eif2a | 5.12 | 4.79 | 0.029 | 0.036 | 0.79 | 0.071 | -1.26 |
| Eif2ak2 | 6.51 | 5.94 | 0.011 | 0.016 | 0.67 | 0.042 | -1.48 |
| Eif2ak3 | 6.34 | 6.29 | 0.012 | 0.013 | 0.96 | 0.406 | -1.04 |
| Eif2ak4 | 6.42 | 6.62 | 0.012 | 0.010 | 1.14 | 0.232 | 1.14 |
| Ern2 | 15.18 | 12.55 | 0.000 | 0.000 | 0.16 | 0.032 | -6.18 |
| Ero1a | 3.15 | 3.48 | 0.113 | 0.090 | 1.26 | 0.389 | 1.26 |
| Fbxo6 | 6.21 | 6.35 | 0.013 | 0.012 | 1.1 | 0.621 | 1.1 |
| Ganab | 3.71 | 3.31 | 0.076 | 0.101 | 0.76 | 0.082 | -1.32 |
| Herpud1 | 2.57 | 3.05 | 0.169 | 0.121 | 1.39 | 0.033 | 1.39 |
| Herpud2 | 6.07 | 5.94 | 0.015 | 0.016 | 0.91 | 0.546 | -1.09 |
| Hsp90b1 | -0.02 | -0.27 | 1.014 | 1.203 | 0.84 | 0.308 | -1.19 |
| Hspa1l | 13.75 | 14.09 | 0.000 | 0.000 | 1.26 | 0.652 | 1.26 |
| Hspa2 | 10.61 | 11.05 | 0.001 | 0.000 | 1.35 | 0.045 | 1.35 |
| Hspa4 | 3.28 | 2.88 | 0.103 | 0.136 | 0.76 | 0.041 | -1.32 |
| Hspa4l | 14.03 | 11.77 | 0.000 | 0.000 | 0.21 | 0.005 | -4.79 |
| Hspa5 | 0.65 | 0.33 | 0.636 | 0.796 | 0.8 | 0.035 | -1.25 |
| Hspb9 | 14.04 | 13.23 | 0.000 | 0.000 | 0.57 | 0.311 | -1.75 |
| Hsph1 | 7.03 | 6.16 | 0.008 | 0.014 | 0.55 | 0.029 | -1.83 |
| Htra2 | 5.31 | 4.94 | 0.025 | 0.033 | 0.78 | 0.136 | -1.29 |
| Htra4 | 11.1 | 10.77 | 0.000 | 0.001 | 0.8 | 0.329 | -1.26 |
| Insig1 | 2.87 | 2.94 | 0.137 | 0.131 | 1.05 | 0.797 | 1.05 |
| Insig2 | 6.86 | 6.87 | 0.009 | 0.009 | 1.01 | 0.931 | 1.01 |
| Manf | 2.28 | 1.96 | 0.206 | 0.256 | 0.8 | 0.028 | -1.24 |
| Mapk10 | 11.98 | 9.68 | 0.000 | 0.001 | 0.2 | 0.001 | -4.92 |
| Mapk8 | 6.49 | 5.85 | 0.011 | 0.017 | 0.64 | 0.017 | -1.56 |
| Mapk9 | 5.81 | 5.6 | 0.018 | 0.021 | 0.86 | 0.165 | -1.16 |
| Mbtps1 | 5.44 | 5.14 | 0.023 | 0.028 | 0.81 | 0.013 | -1.24 |
| Mbtps2 | 6.39 | 6.32 | 0.012 | 0.013 | 0.95 | 0.625 | -1.05 |
| Nploc4 | 4.4 | 3.8 | 0.047 | 0.072 | 0.66 | 0.014 | -1.52 |
| Nucb1 | 4.67 | 4.58 | 0.039 | 0.042 | 0.94 | 0.005 | -1.06 |
| Os9 | 4.02 | 4.09 | 0.062 | 0.059 | 1.05 | 0.546 | 1.05 |
| Pdia3 | 0.49 | 0.25 | 0.712 | 0.839 | 0.85 | 0.239 | -1.18 |
| Pfdn5 | 3.88 | 3.5 | 0.068 | 0.088 | 0.77 | 0.012 | -1.3 |
| Pfdn6 | 3.49 | 3.36 | 0.089 | 0.098 | 0.91 | 0.296 | -1.1 |
| Ppia | -2.45 | -2.56 | 5.452 | 5.883 | 0.93 | 0.280 | -1.08 |
| Ppib | -0.38 | -0.43 | 1.304 | 1.344 | 0.97 | 0.936 | -1.03 |
| Ppic | 6.39 | 6.06 | 0.012 | 0.015 | 0.79 | 0.055 | -1.26 |
| Ppp1r15a | 9.71 | 9.3 | 0.001 | 0.002 | 0.75 | 0.671 | -1.33 |
| Ppp1r15b | 4.41 | 4.26 | 0.047 | 0.052 | 0.9 | 0.371 | -1.11 |
| Prkcsh | 3.95 | 3.78 | 0.065 | 0.073 | 0.89 | 0.337 | -1.13 |
| Rnf139 | 3.38 | 3.48 | 0.096 | 0.090 | 1.07 | 0.148 | 1.07 |
| Rpn1 | 0.68 | 0.54 | 0.626 | 0.688 | 0.91 | 0.287 | -1.1 |
| Scap | 3.62 | 3.55 | 0.082 | 0.086 | 0.95 | 0.648 | -1.05 |
| Sec62 | 1.54 | 1.55 | 0.345 | 0.342 | 1.01 | 0.858 | 1.01 |
| Sec63 | 4.19 | 3.97 | 0.055 | 0.064 | 0.86 | 0.162 | -1.17 |
| Sel1l | 4.65 | 4.54 | 0.040 | 0.043 | 0.93 | 0.521 | -1.08 |
| Vimp | 3.41 | 3.17 | 0.094 | 0.111 | 0.84 | 0.512 | -1.18 |
| Serp1 | 0.51 | 0.3 | 0.704 | 0.814 | 0.86 | 0.194 | -1.16 |
| Sil1 | 6.79 | 6.74 | 0.009 | 0.009 | 0.96 | 0.436 | -1.04 |
| Srebf1 | 9.57 | 8.69 | 0.001 | 0.002 | 0.54 | 0.035 | -1.84 |
| Srebf2 | 9.67 | 9.31 | 0.001 | 0.002 | 0.78 | 0.019 | -1.29 |
| Syvn1 | 5.6 | 5.29 | 0.021 | 0.026 | 0.8 | 0.050 | -1.24 |
| Tcp1 | 2.57 | 2.24 | 0.169 | 0.212 | 0.8 | 0.095 | -1.26 |
| Ube2g2 | 6.86 | 6.62 | 0.009 | 0.010 | 0.85 | 0.079 | -1.18 |
| Ube2j2 | 5.75 | 5.52 | 0.019 | 0.022 | 0.85 | 0.050 | -1.17 |
| Ubxn4 | 4.88 | 4.52 | 0.034 | 0.043 | 0.78 | 0.154 | -1.28 |
| Ufd1l | 5.4 | 5.29 | 0.024 | 0.026 | 0.93 | 0.562 | -1.07 |
| Uggt1 | 5.31 | 4.81 | 0.025 | 0.036 | 0.71 | 0.024 | -1.41 |
| Usp14 | 4.32 | 3.9 | 0.050 | 0.067 | 0.75 | 0.051 | -1.34 |
| Vcp | 2.65 | 2.36 | 0.160 | 0.194 | 0.82 | 0.400 | -1.22 |
| Xbp1 | 3.29 | 3.37 | 0.102 | 0.097 | 1.05 | 0.453 | 1.05 |
| Actb | -1.98 | -2.32 | 3.954 | 4.993 | 0.79 | 0.085 | -1.26 |
| B2m | 0 | 0 | 1.000 | 1.000 | 1 | 0.000 | 1 |
| Hprt1 | 2.03 | 2.13 | 0.244 | 0.228 | 1.07 | 0.239 | 1.07 |
| Ldha | 0.51 | 0.8 | 0.701 | 0.574 | 1.22 | 0.077 | 1.22 |
| Rplp1 | -2.31 | -2.32 | 4.947 | 4.993 | 0.99 | 0.974 | -1.01 |

The housekeeping gene Rplp1 served as control for normalization.

CT: threshold cycle; I/C: IUGR/Control relative expression.
